# Valley Fever on the Rise—Searching for Microbial Antagonists to the Fungal Pathogen *Coccidioides immitis*

**DOI:** 10.3390/microorganisms7020031

**Published:** 2019-01-24

**Authors:** Antje Lauer, Joe Darryl Baal, Susan D. Mendes, Kayla Nicole Casimiro, Alyce Kayes Passaglia, Alex Humberto Valenzuela, Gerry Guibert

**Affiliations:** 1 Department of Biology, California State University Bakersfield, 9001 Stockdale Highway, Bakersfield, CA 93311-1022, USA; Joedarryl.Baal@ucsf.edu (J.D.B.); susiedmorley@gmail.com (S.D.M.); kaylacasi@gmail.com (K.N.C.); akayes@csub.edu (A.K.P.); avale043@ucr.edu (A.H.V.); 2 Monterey County Health Department, 1270 Natividad, Salinas, CA 93906, USA; guibertg@gmail.com

**Keywords:** *Coccidioides immitis*, Valley Fever, biocontrol, microbial antagonists, challenge assays, arid soils

## Abstract

The incidence of coccidioidomycosis, also known as Valley Fever, is increasing in the Southwestern United States and Mexico. Despite considerable efforts, a vaccine to protect humans from this disease is not forthcoming. The aim of this project was to isolate and phylogenetically compare bacterial species that could serve as biocontrol candidates to suppress the growth of *Coccidioides immitis*, the causative agent of coccidioidomycosis, in eroded soils or in areas close to human settlements that are being developed. Soil erosion in *Coccidioides* endemic areas is leading to substantial emissions of fugitive dust that can contain arthroconidia of the pathogen and thus it is becoming a health hazard. Natural microbial antagonists to *C. immitis*, that are adapted to arid desert soils could be used for biocontrol attempts to suppress the growth of the pathogen in situ to reduce the risk for humans and animals of contracting coccidioidomycosis. Bacteria were isolated from soil samples obtained near Bakersfield, California. Subsequently, pairwise challenge assays with bacterial pure cultures were initially performed against *Uncinocarpus reesii*, a non-pathogenic relative of *C. immitis* on media plates. Bacterial isolates that exhibited strongly antifungal properties were then re-challenged against *C. immitis*. Strongly anti-*C. immitis* bacterial isolates related to *Bacillus subtilis* and *Streptomyces* spp. were isolated, and their antifungal spectrum was investigated using a selection of environmental fungi.

## 1. Introduction

Valley Fever, also known as coccidioidomycosis, is an infection caused by the ascomycete fungal pathogens *Coccidioides* spp. The dimorphic *C. immitis* and *C. posadasii* are endemic to soils of American deserts and semi-arid regions where they can live as soil saprophytes. *C. immitis* is thought to be endemic to the Southern San Joaquin Valley, whereas *C. posadasii* is predominantly localized in Southern Arizona, but it has also been detected in other states of the Southwestern United States, such as Texas, Nevada, New Mexico, and areas in Mexico and South America [1,2]. Recently, *C. immitis* was isolated from Washington State and the expansion of its range due to climate change is being discussed [3,4,5]. Although *Coccidioides* spp. are considered to be maintained in rodent reservoirs [6,7], arthroconidia of the pathogen can become airborne when they grow as a soil saprophyte following soil disruption. When inhaled, these dormant forms of the pathogen can infect the lung of humans and animals, primarily mammals [8,9].

Epidemiological studies documented that the number of reported cases of coccidioidomycosis in the United States increased from 1200 in 1995 to more than 20,000 in 2011, including about 5000 cases in California, and more than 3000 documented deaths were noted nationwide where the disease was an underlying or contributing cause [10]. In 2016 and 2017, prevalent cases reached a similar number [11,12]. Coccidioidomycosis is widely underdiagnosed and therefore underreported. It is estimated that more than 200,000 cases of coccidioidomycosis occur annually in the United States alone [13,14]. Kern County in the Southern San Joaquin Valley of California is a well-known highly endemic area for *C. immitis* with a generally high annual incidence of more than 2000 cases in 2016 (42% of all reported cases in California) [12]. With growing numbers of elderly and immunosuppressed persons in the United States, the number of coccidioidomycosis related deaths will probably increase, resulting in higher costs to the health care system and increased human suffering [15]. Given the significant disease burden, it is surprising that coccidioidomycosis is still considered an ‘orphan disease’ [16,17]. Furthermore, it should be noted that the development of a vaccine to protect humans from coccidioidomycosis has been unsuccessful so far, despite considerable efforts and promising initial results [18].

Although much is known about the physiology of *C. immitis* and how it causes disease [19,20], many fundamental gaps in our understanding of the ecology of this pathogenic fungus persist. It is known that the pathogen is adapted to loamy sands with elevated salt concentrations, and that it is able to withstand high surface temperatures (>40 °C) [9,21], unlike other microorganisms that share the Lower Sonoran Life Zone with arid to semi-arid soils. There are few published data available about the distribution of *C. immitis* growth sites in California [22,23] and even fewer on the diversity of bacteria, fungi, and other microorganisms in these types of soils [24,25]. *C. immitis* and also *C. posadasii* growth sites are often detected in nutrient poor, arid soils with increased pH and electrical conductivity, extreme environments where surface soil temperature easily reach > 60 °C during the summer (e.g., alkali sinks, dry lakes, and salt bush areas). In these desert environments, the pathogen likely encounters less antagonism from other soil microbes [9]. It is unknown how soil microorganisms interact with *Coccidioides* spp. in their natural habitat and whether there is any antibiosis or how the microbial community, including the pathogen, reacts to seasonal changes or to human influence (e.g., agriculture, pollution, and disturbance of soil due to construction). Egeberg et al. [25] showed that *C. immitis* is able to grow on Sabouraud medium supplemented with 8% NaCl or CaCl_2_, in contrast to two bacterial and one fungal antagonist.

In the complex soil environment, microbial diversity and the presence or absence of potential plant, animal, and human pathogens are influenced by soil physical and chemical parameters, but also factors such as seasonal influences and climate change, soil disturbance, diversity of plant growth, presence or absence of root exudates, and pollutants [26,27,28,29,30,31] impact microbial diversity. A very significant factor is also the interaction between microbial organisms through synergism and antagonism [25,32]. Microbial antagonism is common in soil and might explain the absence of *Coccidioides* spp. in soils that theoretically could support their growth based on certain physical and chemical parameters. Biological parameters, such as the presence of plants, are important for the establishment of microbial populations as well. The influence of plant root secretions on soil borne microbial communities as a powerful selective force has been confirmed [33,34,35,36,37]. Antimicrobial compounds have repeatedly been implicated in the antagonism of *Streptomyces* and *Bacillus* species against soil fungi. Some strains of *Bacillus subtilis* have been described as antifungal bacterial agents. A list of antifungal substances produced by *Streptomyces*, *Bacillus* and other bacteria has been published [38], and some may also inhibit the growth of *Coccidioides* spp., but this has never been investigated. In agriculture, bacterial antagonists have been successfully used to protect plants from certain plant pathogens, especially fungi [39,40,41,42], but biocontrol of fungi that can cause disease in humans is uncommon. Fungal-bacterial interactions (FBI) influence microbial community diversity and have been studied predominantly in terrestrial systems [43,44,45,46]. Only two microbial strains that showed antagonistic properties to *C. immitis* in vitro have been identified in the past: a strain related to *Bacillus subtilis* and a strain of *Penicillium janthinellum* [25].

Our project focused on the isolation and phylogenetic comparison of anti-*C. immitis* bacterial isolates from loamy soils collected Northeast and Southwest of Bakersfield, California, by performing pairwise FBI on artificial media plates. The aim of this study was to obtain anti-*C. immitis* bacterial isolates that are heat- and salt tolerant and adapted to grow in arid soils. These bacterial isolates could be candidates for the development of a biocontrol method to suppress the pathogen in natural soils on land destined for development without negatively influencing most other soil fungi.

## 2. Materials and Methods

### 2.1. Sampling Sites

**Figure 1:**
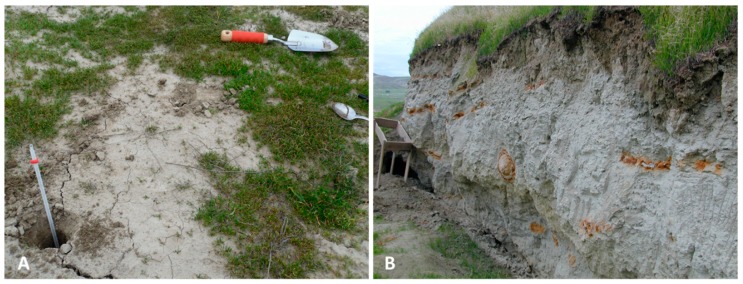
(**A**) Soils at site A (Cole’s Levee Road [CLR]) appearing dry and low in organic matter. (**B**) Site B (near the California Living Museum [CALM]) showing evidence of fossil digging.

For the isolation of potential anti-*C. immitis* bacterial species, two sampling sites (Figure 1) were chosen that were similar in soil texture and physical and chemical parameters to sites identified in a previous study to support the growth of *C. immitis*. Site A is a non-agricultural field located Southwest of Bakersfield close to the Buena Vista Aquatic Recreational Area that has tested positive for the presence of the pathogen in two consecutive years (data not shown). Soil physical and chemical parameters obtained from the websoilsurvey database of the United States Department for Agriculture (USDA) are similar to other sites around Kern County where the pathogen has been detected [20]. Site B is close to a *C. immitis* positive site known as Sharktooth Hill [21,47] and is located Northeast of Bakersfield, near the California Living Museum (CALM). Both sites are partially covered with different species of grasses (predominantly invasive *Bromus* spp.) and other native and non-native annuals. Site descriptions and soil parameters are listed in Table 1.

Environmental fungi were isolated from outside air at the California State University Bakersfield (CSUB) campus.

### 2.2. Soil Sampling

Soil samples (~20 g, 5–7 cm depth) were collected using aseptic techniques and placed into sterile containers (Thermo Scientific* Samco* Wide-Mouth Bio-Tite* Specimen Containers, Fisher Scientific, Pittsburgh, PA, USA). They were transported to the laboratory on ice and processed for bacterial isolation on the same day.

### 2.3. Isolation of Bacteria and Fungi

To obtain bacterial isolates, soil samples were diluted 1:100,000 in a 10-fold dilution series, and aliquots of 100 µL were plated onto low nutrient R2A medium (Difco) that was supplemented with 10% soil extract (SE) (3 replicate plates). Soil extract was produced by autoclaving 200 g of soil from the original sampling sites with 200 mL of distilled water. After autoclaving, the soil slurry settled overnight, and the supernatant was harvested and used as medium supplement. Soil extract was included to provide the growing microorganisms with a diversity of nutrients and trace elements from their natural habitat. It has been shown in previous research that the addition of soil extract to growth media can significantly increase the diversity of growing bacteria by providing additional nutrients and growth factors [48]. The plates were incubated for two weeks at room temperature under aerobic conditions. All colony morphology types that resembled spore forming *Bacillus* and *Streptomyces* related species were transferred onto new media plates until pure cultures were obtained. The Gram behavior of the bacterial isolates was determined by using the Gram stain method [49]. We focused on Gram positive spore formers, because these species are more likely to withstand harsh environmental conditions compared to most Gram-negative species. Spore-forming microbes can easily be harvested and stored and are therefore good candidates for biocontrol attempts. 

To obtain fungal isolates from airborne spores, R2A+SE plates were exposed to the outside air at the CSU Bakersfield campus for 10 min. These plates were also incubated for up to 2 weeks at room temperature under aerobic conditions. Different fungal colonies were re-streaked onto new media plates until pure cultures were obtained.

### 2.4. Challenge Assays

All bacterial pure cultures, isolated from our soil samples, were initially screened for antifungal activities against *Uncinocarpus reesii* which has been considered a non-pathogenic strain of *C. immitis* by some researchers [50,51,52]. All anti-*U. reesii* bacterial isolates were subsequently challenged for anti-*C. immitis* activity at the Monterey Public Health Laboratory in Salinas, California. Fungal colonies were grown in the center of R2A+SE plates and allowed to grow to about 1 cm diameter at 30 °C, before two bacterial isolates were inoculated at either site of the fungus in a simple streak (distance 1 cm). Plates were incubated for one week at 30 °C and then evaluated for a visible zone of inhibition between the fungus and the bacterial isolates. The challenge assays were scored visually as follows:

1. Strongly antifungal: an inhibition zone of several mm appeared between fungi and bacteria. The fungus was unable to overgrow the bacteria.
2. Weakly antifungal: the density of the fungal mycelium was lower toward the bacterial area, whereas all bacteria free space on the plate was covered with a thick fungal mycelium. The fungal growth adjacent to the bacterial growth was also slowed down considerably.
3. Not antifungal: the whole plate was evenly covered with fungal hyphae [53,54].

All anti-*C. immitis* bacterial isolates, from the screening described above, were then challenged for antagonistic effects against different environmental fungi using the method described above to investigate the spectrum of antagonism (broad or narrow). The mechanisms of antibiosis were not investigated.

*U. reesii* was purchased from the American Type Culture Collection (ATCC, # 34533), and a *C. immitis* isolate was obtained from the laboratory of the County of Kern Public Health Services Department, Bakersfield, CA (patient specimen KC12672). A PCR fragment (475 bp) of this isolate obtained with primer pair EC3/EC100 (see Section 2.6) was 99% related to an 18S rDNA fragment from *C. immitis* in the GenBank nucleotide database (Accession # MH863096) of the National Center of Bioinformatics (NCBI).

### 2.5. Heat and Salt Tolerance of Anti-Coccidioides Bacterial Isolates

All anti-*C. immitis* bacterial isolates obtained in this study were investigated for their ability to tolerate extreme environmental conditions, such as increased salt concentrations and increased temperature. The bacteria were grown on R2A+SE supplemented with 10% and 20% sodium chloride or with 0.5% borax (disodium tetraborate), a compound that is often found in *Coccidioides* endemic habitats in California and which is known for its antimicrobial properties [55]. These plates were incubated at 30 °C under aerobic conditions. Furthermore, the bacterial isolates were investigated for their abilities to tolerate 40 °C, 50 °C, and 63 °C on R2A+SE medium. All plates were incubated for one week and then evaluated for growth.

### 2.6. DNA Extraction from Bacterial and Fungal Pure Cultures and PCR

The phylogenetic relationship of bacterial isolates was investigated using DNA extraction followed by PCR targeting the ribosomal gene. DNA from anti-fungal bacterial isolates and from environmental fungi was extracted with the MoBio Microbial DNA isolation kit (MoBio, Solana, CA, USA) according to the manufacturer’s protocol. Fragments of the 16S rRNA gene from bacterial isolates resembling members of the actinomycetes based on visual observations were amplified using primer pair 243F/1378R following the procedure described in [56]. Bacterial isolates that did not resemble strains of actinomycetes were amplified with primer pair 8F/1492R [57,58]. DNA from fungal isolates was amplified using primer pair NSA3/NLC2 [59]. Amplification reactions were carried out in a 25 µL reaction volume containing 12.5 µL 2x GoTaq Green MasterMix (Promega, Madison, WI), 1.5 µL of each primer (250 nmol), 1.5 µL of DNA template (10-100 nmol), 7.5 µl of sterile ddH_2_O using a C1000 Touch Thermal Cycler (BioRad, USA) and were based on the protocols referenced earlier. Briefly, for primer pair 243F/1378R an initial denaturation step of 5 min at 94 °C was followed by 35 cycles which consisted of 1 min at 94 °C, 1 min at 63 ºC, and 1 min at 72 °C with a final elongation at 72 °C for 10 min. For primer pair 8F/1492R an initial denaturation at 94 °C for 4 min was followed by 35 cycles which consisted of 30 sec at 94 °C, 30 sec at 50 °C and 1 min at 72 °C, followed by a final elongation at 72 °C for additional 10 min. The amplification protocol using fungal DNA targeting for 18S rDNA followed the steps as described in detail by Martin and Rygiewicz [59]. The correct size of all PCR products was verified on 2% agarose gels in 1X Tris, borate, EDTA (TBE) buffer with a PCR marker for comparison (exACTGene, Fisher Scientific, Pittsburgh, PA). Gels were stained with SYBRsafe (Invitrogen, Carlsbad, CA, USA) and documented using the Universal Hood II Gel documentation system (BioRad, Hercules, CA, USA).

### 2.7. Sequencing and Phylogenetic Analysis of rDNA Fragments

In preparation of sequencing, PCR products from microbial pure cultures were purified using the Qiagen PCR purification kit (Qiagen, USA) according to the manufacturer’s instructions. All rDNA fragments were sequenced at the Interdisciplinary Center for Biotechnology Research (ICBR) at the University of Florida. All retrieved sequences were compared to entries in the nucleotide database GenBank using the Basic Local Alignment Search Tool (BLAST) (www.ncbi.nlm.nih.gov). Finally, a phylogenetic tree was assembled which included bacterial rDNA sequences from anti-fungal bacterial isolates together with their closest matches in the GenBank nucleotide database with support of the software MEGA 7 [60].

## 3. Results

### 3.1. Isolation of Bacterial and Fungal Pure Cultures

Spore forming bacterial species such as *Streptomyces* spp. and *Bacillus* spp. were recognizable on the original isolation plates based on their colony morphology and made up a considerable fraction of the overall colony morphology types. The plate count on R2A+SE medium revealed approximately 15.6 × 10^5^ cfu/mL, predominantly bacteria. About 4 % of the colonies visually represented *Streptomyces* spp. or related species based on their colony morphology (rigid and round colonies, powdery surface due to spore formation, earthy smell, and release of pigments into the surrounding medium). Fungal species were observed on the plates as well, predominantly belonging to the genus *Penicillium*, as revealed by colony color (mostly turquoise green) and microscopy of conidia characteristic for this genus [61]. *Coccidioides* spp. did not grow on any plates.

Overall, 100 bacterial isolates were obtained in pure culture from both sampling sites (60 isolates from sampling site A [CLR], and 40 from site B [across CALM]). As mentioned earlier, we focused on the isolation of Gram positive, spore forming bacterial species such as *Streptomyces* and *Bacillus* species, because these organisms are better adapted to harsh conditions in their natural habitat because of their ability to form spores. Gram negative, non-spore forming species were also part of the natural bacterial community in these soils (presented by predominantly pink colonies on original plates). Furthermore, 13 fungal isolates were obtained from the air at CSUB campus.

**Figure 2:**
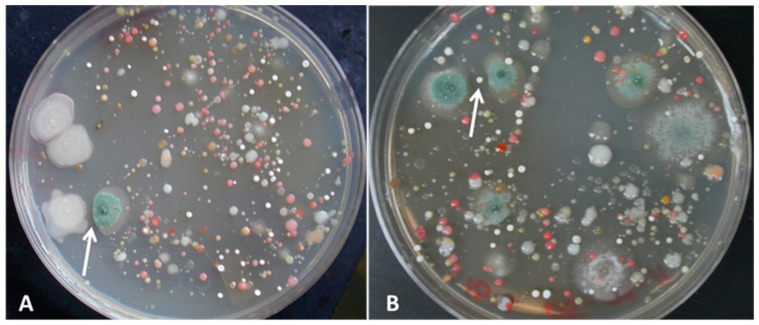
Visible zone of inhibition between bacterial colonies and fungal colonies (samples from site 1 [CLR]). (**A**) A large bacterial colony related to *Bacillus* sp. is inhibiting the growth of a *Penicillium* sp. Small white colonies were identified as members of the *Streptomyces* genus which indicates that these bacteria are among the dominant members of the cultivable soil microbiota at our sampling sites. Results for site 2 were similar (not shown). (**B**) A small bacterial colony identified as *Streptomyces* sp. is inhibiting a *Penicillium* sp. The zones of inhibition are indicated by a white arrow.

Antagonistic effects between some bacterial colonies and some fungi were observed on the original media plates (Figure 2).

### 3.2. Challenge Assays

**Figure 3:**
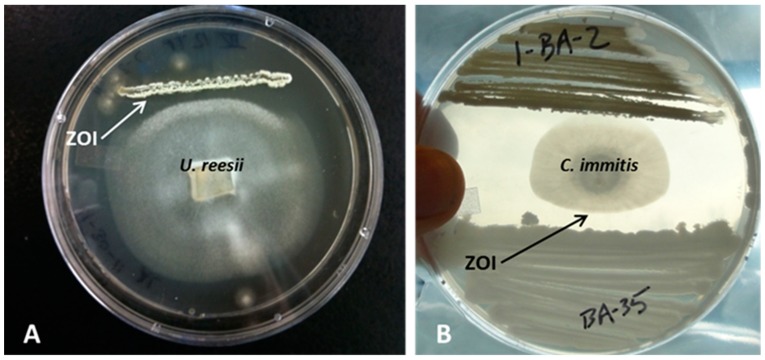
Results of challenge assays on R2A+SE medium. (**A**) Bacterial isolate # F, closely related to *Streptomyces anulatus* (AB184199), showed antifungal activity against *U. reesii*. A distinct zone of inhibition (ZOI) is visible. (**B**) Isolate # BA-35, closely related to *Bacillus subtilis* (JN641290), showed antifungal activity against *C. immitis*. No anti-*C. immitis* activity was observed for isolate I-BA-2 (a *Streptomyces* sp. that was antifungal against *U. reesii*).

Of the 100 bacterial cultures obtained from both sampling sites, 35 inhibited the growth of *U. reesii* in challenge assays on R2A+SE. These isolates predominantly belonged to different species of *Streptomyces*, but also some members of the genus *Bacillus* showed anti-*U. reesii* properties. From these 35 anti-*U. reesii* bacterial isolates, eight inhibited *C. immitis* (Table 2). These anti-*C. immitis* bacterial isolates were subsequently challenged against different environmental fungi identified as members of the Pleosporales, Hypocreales, Chaetothyriales and Eurotiales. Differences in the spectrum of their anti-fungal effects were observed. Generally, bacterial isolates related to the Bacillaceae showed a broader spectrum of antifungal behavior compared to members of the Actinobacteria. Some of the isolated *Streptomyces* species were only antifungal against the Onygenales *U. reesii* and *C. immitis*. Examples of challenge assay results are presented in Figure 3.

### 3.3. Phylogenetic Relationships of Bacterial Isolates with Anti U. reesii and Anti-C. Immitis Properties

**Figure 4:**
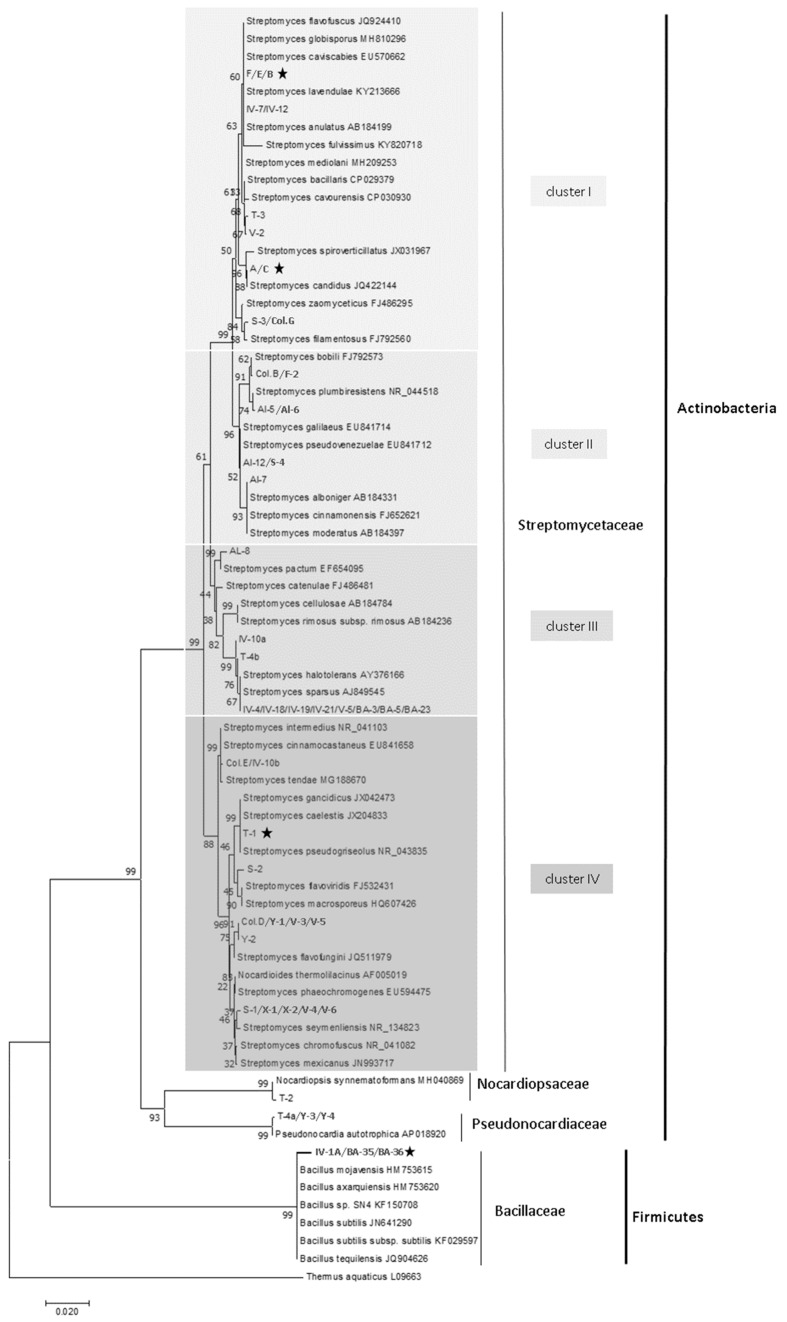
Phylogenetic relationship between Gram positive antifungal bacterial isolates obtained in this study and other species belonging to the genera *Streptomyces*, *Pseudonarcdia* and *Bacillus* based on a ~1100 bp fragment of the 16S rRNA gene. Bacterial isolates belonging to the Streptomycetaceae can be divided into four main clusters with closest matches in the GenBank nucleotide database. The evolutionary history was inferred using the Neighbor-Joining method [62]. The percentage of replicate trees in which the associated taxa clustered together in the bootstrap test (100 replicates) are shown next to the branches [63]. The tree is rooted using a sequence from *Thermus aquaticus* as outgroup. A star indicates bacterial isolates which were anti-*U. reesii* and anti-*C. immitis*. Evolutionary analyses were conducted in MEGA7 [64].

We were able to phylogenetically describe all anti-fungal bacterial species by sequencing about 1100 bp of the 16S rRNA gene obtained with primer pairs 243F/1378R or 8F/1492R. The majority (87.5 %) of the bacterial isolates belonged to different members of the genus *Streptomyces*. The fragment size of 1100 bp did not allow us to identify our bacterial isolates to the species level but it was sufficient to phylogenetically compare them. The phylogenetic analysis revealed four main clusters within the family Streptomycetaceae with bacterial isolates related to *S. flavofuscus* and *S. filamentosus* (cluster I), *S. plumbiresistens* and *S. moderatus* (cluster II), *S. catenulae* and *S. sparsus* (cluster III), *S. intermedius* and *S. mexicanus* (cluster IV). Strongly anti-*C. immitis* isolates were closely related to *S. candidus* (cluster I). The most prevalent *Streptomyces* isolates with anti-*U. reesii* properties were related to *S. seymenliensis* (cluster IV), but they did not possess anti-*C. immitis* properties. The remaining 12.5% of the bacterial isolates with antifungal behavior were members of the genera *Bacillus*, *Pseudonocardia* and *Nocardiopsis*. The strongly anti-*C. immitis* isolates that were phylogenetically related to *Bacillus* spp. were closely related to *B. subtilis* and other Bacillaceae that had been isolated from the Mojave Desert Mexico, and Saudi Arabia, such as *B. mojavensis*, *B. tequilensis*, and *B. axarquiensis*. A phylogenetic analysis of all 16S rRNA sequences together with closest matches from the GenBank database is presented in Figure 4. The Genbank accession numbers of anti-*Coccidioides* bacterial rDNA sequences are KF638402-KF638416.

### 3.4. Heat and Salt Tolerance of Anti-Coccidioides Bacterial Isolates

The bacterial isolates varied in their response to different growth conditions in the laboratory. Many isolates tolerated temperatures up to 50 °C, but only one isolate, related to a *Bacillus* spp., grew at 63 °C. Many *Streptomyces* species formed colonies on R2A+SE medium supplemented with 0.5% borate, in contrast to *Bacillus* species. Not all bacterial isolates with anti-*C. immitis* activities tolerated these extreme conditions (Table 3).

## 4. Discussion

Interest in biological control has recently increased due to public concerns regarding the use of chemicals in the environment, especially in agriculture where it is used to suppress plant pathogens and to increase harvest. The need to find non-chemical alternatives has fueled investigation on natural microbial antagonists to a variety of pathogens [65,66,67].

In our research, we did not anticipate isolating novel antibiotic producing bacterial species or antifungal compounds. Instead, the focus of our efforts was to identify suitable members of the soil microbial community that show potential to inhibit the pathogenic fungus *C. immitis* and are also able to withstand adverse conditions in alluvium-derived, arid, and salinic soils that are typical for *Coccidioides* spp. habitats in the Southwestern United States and Mexico.

In this study, several *Streptomyces*, *Pseudonocardia*, *Nocardiopsis*, and *Bacillus* species with anti-*C. immitis* activity were isolated from two different locations near Bakersfield, Kern County, California. A selection of these bacterial isolates was further investigated regarding their antifungal spectrum against a variety of other fungal species to assess their suitability as potential selective biocontrol agents against the pathogen *C. immitis*. Among these bacterial isolates was only one (IV-1A, phylogenetically related to *B. subtilis*) that showed growth above 50 °C and tolerance to increased salinic conditions, characteristics that allowed it to thrive when other soil microbes are inhibited. However, this bacterial isolate inhibited most other soil fungi in pairwise FBI in this study and therefore it might be harmful for beneficial soil fungi. Several antifungal isolates related to *Streptomyces* species were able to tolerate salinic conditions as well but most stopped growing when temperature was increased to 50 °C (exception: isolate T-4b). Among these isolates were several that showed a narrow spectrum of antibiosis, some only inhibited *C. immitis*, which makes them good candidates for selective biocontrol. Based on early laboratory studies, it has been suspected that *Coccidioides* spp., as weak competitors, favor environments with reduced diversity, such as alkaline and salinic soils where fewer potential antagonists are established [24,68,69].

Arid and semi-arid environments, such as the soils investigated in this study, are often dominated by spore formers such as fungi and bacteria that are related to actinomycetes and *Bacillus* species which can survive heat stress and desiccation in their dormant forms. Different *Streptomyces* species have been found to be associated with arid soils, often established within the rhizosphere of certain desert plants where they contribute to overall plant growth and health [34,70,71,72]. We have shown in this study that *Streptomyces* spp. were abundant in soils of our study sites which are known to support the growth of *C. immitis* [21,24,68], and which might explain its spotty distribution. 

Various methods to detect fungal growth inhibition *in vitro*, such as the pairwise challenge assays performed in this study, have been successfully developed and applied in the past [53,54,73,74]. In vitro screening assays, such as these FBI, can be useful in selecting microbial antagonists for in vivo experiments. However, direct extrapolations from results obtained by these assays to different levels of complexity such as ecosystems should be made carefully. Because of the ‘great plate count anomaly’ [75], and the selection of only one type of medium in this study, we were not able to isolate all microbial species with anti-*C. immitis* properties that reside in these types of soils. However, we were able to obtain several anti-*C. immitis* bacterial isolates within the Actinobacteria and Firmicutes (Figure 4). Only two microbial strains that are antagonistic to *C. immitis* have been described in the past: one strain related to *Bacillus subtilis*, and a second related to *Penicillium janthinellum*. However, neither organism inhibited *C. immitis* in laboratory experiments when incubation conditions reached 40 °C and when salinity was increased, mimicking environmental conditions of the soil during the summer months [24]. Furthermore, it should be noted that fungal inhibition can be lost by interactions with other microbes in a complex soil environment [45].

Antifungal activity is a relatively common characteristic among soil bacteria and fungi that compete for space and nutrients. This confers an ecological advantage in environments that support the growth of mixed microbial communities. Bacteria that show antifungal capabilities in vitro may or may not be active antagonists in the soil, while those being non-antifungal in vitro are generally also inactive in their natural environment, as has been shown by other researchers [46,76]. In addition, soil temperature, pH, moisture, and application of phosphate fertilizers are known factors that influence antibiotic production and activity [77,78,79,80]. An established microbial organism isolated from a site that could also support the growth of *C. immitis*, is likely a more suitable candidate for a biocontrol approach to inhibit the growth of the pathogen, compared to an exotic organism that may or may not adapt to the existing chemical and physical conditions in a particular soil. Furthermore, to support the establishment of antifungal biocontrol agents, Cretoiu et al. [81] showed that chitin amendment supported the growth of antifungal bacteria that produced the enzyme chitinase and increased soil suppressiveness toward plant pathogens. Therefore, amendment of soils to be treated with nutrients and growth factors to support the antagonist to the pathogen should be considered for a successful biocontrol approach.

Coccidioidomycosis is a well-documented occupational hazard in California. Furthermore, epidemiological studies have established that the burden of this disease is a significant problem in prisons located within endemic areas of California. Microbial antagonists to *C. immitis*, such as *B. subtilis* or *Streptomyces* spp., could be considered in a biocontrol approach to suppress the growth of the pathogen in endemic areas of risk (e.g., construction sites and areas surrounding prisons) [82,83]. Choosing a mixture of anti-*Coccidioides* microbial species that are members of the microbial community (which naturally reside in these types of soils and enhance their presence) in order to keep negative impacts on other members of the soil microbial community and local plants and animals to a minimum, could be proposed. Any risks need to be carefully investigated before biocontrol procedures are attempted in areas outside controlled conditions in the laboratory. *Bacillus* spp. and *Streptomyces* spp. like those isolated in this study can be used as probiotics in future studies to suppress the growth of *C. immitis* in soils of California. However, it has to be confirmed in situ that these antagonists will also be effective against the pathogen in its natural habitat and that the benefit of a biocontrol approach will outweigh any potential negative influences on other soil inhabitants, as well as plants, animals, and humans who reside in the area [84,85]. Furthermore, it should be considered that *Coccidioides* strains might vary in their ability to withstand microbial antagonism. Ideally, several *Coccidioides* isolates should be obtained from sites to be treated to confirm the antifungal abilities of any microbial antagonist to the pathogen before tests are conducted *in situ*.

## 5. Conclusions

Spraying water to reduce dust prior and during constructions in arid and semiarid environments is common but could enhance the growth of *Coccidioides* spp. in the soil. Adding microbial antagonists, such as those described in this study, to the water and spraying this mixture several weeks prior to the development of an area might be an alternative method to reduce the risk of contracting coccidioidomycosis. Furthermore, areas near schools and recreation sites that are known growth areas of the pathogen could be treated in early spring to suppress the growth of *Coccidioides* spp.

Our results might be of interest to other researchers who focus on different soil borne pathogens that cause similar infectious diseases as Valley Fever, such as blastomycosis and histoplasmosis which, together with coccidioidomycosis, are the major pulmonary mycoses of humans, because these fungal pathogens can also grow as soil saprophytes [86,87]. Future work with anti-*Coccidioides* bacterial isolates may include the characterization of genes involved in anti-fungal production and investigations on the mode of action of these anti-fungal compounds.

## Figures and Tables

**Table 1:** Soil physical and chemical parameters obtained from the United States Department for Agriculture (USDA) websoilsurvey database for our two sampling sites and reference site.

	Sampling Sites		Reference Site
	Site A (Cole’s Levee Rd.)	Site B (across CALM)	Sharktooth Hill
**Soil Parameters**			
coordinates	119° 13″ 60.0′ W, 35° 14″ 08.0′ N	118°513″ 14.1′ W 35° 25″ 50.3′ N	118° 54″ 37.0′ W, 35° 28″ 20.0′ N
vegetation	grasses and herbs (native and non-native)	grasses and herbs (native and non-native)	grasses and herbs (native and non-native)
soil type	Garces loam	Chanac clay loam	Pleito-Trigo-Chanac complex
landform	fan remnants	fan remnants	fan remnants/stream terraces
parent material	alluvium derived from granitoid	alluvium derived from mixed	alluvium derived from mixed
(soil map unit symbols)	180	130	205
drainage class	well drained	well drained	well drained
maximum salinity (mmhos/cm)	8–16	0–2	0–2
**Physical Parameters**			
Surface texture	clay loam	clay loam	gravelly clay loam
Clay (%)	25.5	31	33.5
Silt (%)	36.5	33.6	36.5
Sand (%)	38	35.4	30
Available water capacity (cm/cm)	0.21	0.17	0.16
Available water supply (0-25 cm)	5.04	4.25	3.69
Organic matter (%)	0.98	0.75	1.5
Water content (15 bar)	16.7	18.2	17.2
Water content (1/3 bar)	30.9	30.1	29.3
Sat. hydraulic conductivity (Ksat) (micrometers/s)	8.37	9	2.82
**Chemical Parameters**			
pH	8.5	7.9	7.8
CaCO_3_ (%)	3	3	0
Cation exchange capacity (CEC7) (milliequivalents/100 grams)	20.6	24.4	24.3
Gypsum (%)	0	0	0
Sodium adsorption ratio (SAR)	2	0	0
Electrical conductivity (decisiemens/m)	5	0	0.5

**Table 2:** Challenge assays of bacterial isolates against environmental fungal isolates revealed differences in antifungal spectra.

Isolate #	Fungal ID	GenBank Accession # or ATCC #	Similarity (%)	Challenge Assays: Bacteria against Fungi							
				Actinobacteria					Firmicutes		
				IV-4	IV-7	IV-18	BA-3	T-4b	IV-1A	BA-35	BA-36
KVF4	*Alternaria alternata*	AY154682	96	neg	neg	neg	neg	neg	**pos**	**pos**	**pos**
LDF1	*Aspergillus versicolor*	KJ082097	99	neg	neg	**pos**	neg	neg	neg	**pos**	**pos**
LDF2	*Aspergillus keveii*	MF004311	98	neg	neg	neg	neg	neg	**pos**	**pos**	**pos**
ZMF2	*Lewia infectoria*	AY154691	98	neg	**pos**	neg	neg	neg	**pos**	**pos**	**pos**
AKF4	*Aspergillus keveii*	MF004311	98	neg	neg	neg	neg	neg	**pos**	**pos**	**pos**
AKFB	*Penicillium citrinum*	KM491892	99	neg	**pos**	neg	neg	neg	**pos**	neg	**pos**
482-2	*Penicillium gladioli*	DQ339568	92	neg	neg	neg	neg	neg	neg	**pos**	**pos**
426-1	*Penicillium gladioli*	DQ339568	92	neg	neg	neg	neg	neg	**pos**	**pos**	**pos**
435-2	*Penicillium gladioli*	DQ339568	92	neg	neg	neg	neg	neg	**pos**	**pos**	**pos**
N26-6	*Peniophora sp.*	HQ608067	98	neg	neg	neg	neg	neg	neg	neg	neg
ZMF-1	*Fusarium proliferatum*	LT841264	97	neg	neg	neg	neg	neg	**pos**	**pos**	**pos**
AKF7	*Fusarium accuminatum*	KJ019024	98	neg	neg	neg	neg	neg	neg	neg	neg
*U. reesii*	*Uncinocarpus reesii*	ATCC34534	100	**pos**	**pos**	**pos**	**pos**	**pos**	**pos**	**pos**	**pos**
*C. immitis*	*Coccidioides immitis*	MH863096	99	**pos**	**pos**	**pos**	**pos**	**pos**	**pos**	**pos**	**pos**

**Table 3:** Colony morphologies and closest matches in the GenBank nucleotide database, as well as tolerance to increased temperature, borate and NaCl concentrations for all bacterial isolates investigated are shown.

Isolate ID	Colony Morphology on R2A+SE	Closest Match in GenBank with Similarity (%) and Accession #	R2A+SE, Incubated at Different Temperatures			R2A+SE, Supplemented with Salt		
			40 °C	50 °C	63 °C	Borate (0.5%)	NaCl (10%)	NaCl (20%)
BA-35	large, white-tan, dull, flat, irregular margin	*Bacillus subtilis*, 99%, CP024961	**pos**	**pos**	neg	neg	**pos**	neg
BA-36	large, white-tan, dull, flat, irregular margin	*Bacillus subtilis*, 99%, CP024961	**pos**	**pos**	neg	neg	**pos**	neg
IV-1A	large, white-tan, dull, flat, irregular margin	*Bacillus subtilis*, 99%, CP024961	**pos**	**pos**	**few colonies**	neg	**pos**	neg
V-2	medium size, grey	*Streptomyces cavourensis*, 98 %, CP030930	**pos**	neg	neg	neg	neg	neg
T-3	large size, white	*Streptomyces cavourensis*, 97 %, CP030930 / *Streptomyces bacillaris*, 98%, CP029379	**pos**	neg	neg	**pos**	neg	neg
IV-10a	large size, white	*Streptomyces halotolerans*, 97%, AY376199	**pos**	neg	neg	neg	neg	neg
IV-7	medium size, purple	*Streptomyces lavendulae*, 99%, KY213666	**pos**	neg	neg	neg	neg	neg
IV-12	medium size, white	*Streptomyces fulvissimus*, 99%, KY820718	**pos**	neg	neg	neg	neg	neg
T-4b	large size, white	*Streptomyces halotolerans*, 97%, AY376199	**pos**	**pos**	neg	neg	neg	neg
V-5	small size, white	*Streptomyces sparsus*, 97%, KM999546	**pos**	neg	neg	**pos**	**pos**	neg
IV-4	medium size, white	*Streptomyces sparsus*, 97%, KM999546	**pos**	neg	neg	**few colonies**	**pos**	neg
IV-18	medium size, white	*Streptomyces sparsus*, 98%, KM999546	**pos**	neg	neg	**pos**	**pos**	neg
IV-21	medium size, white	*Streptomyces sparsus*, 99%, KM999546	**pos**	neg	neg	**few colonies**	**pos**	neg
IV-19	medium size, white	*Streptomyces sparsus*, 99%, KM999546	**pos**	neg	neg	**pos**	**pos**	neg
BA-3	small size, white	*Streptomyces sparsus*, 98%, KM999546	**pos**	neg	neg	**pos**	**pos**	neg
BA-5	medium size, white	*Streptomyces sparsus*, 99%, KM999546	**pos**	neg	neg	**pos**	**pos**	neg
BA-23	medium size, white	*Streptomyces sparsus*, 99%, KM999546	**pos**	neg	neg	**pos**	**pos**	neg
Y-2	medium size, purple	*Streptomyces flavofungini*, 94%, JQ511979	**pos**	neg	neg	neg	neg	neg
Col.B	small size, white	*Streptomyces bobili*, 99%, FJ792573	**pos**	neg	neg	neg	neg	neg
T-2	large size, white	*Nocardiopsis synnemataformans*, 99%, MH040869	**pos**	neg	neg	**pos**	neg	neg
T-4a	tiny size, white	*Pseudonocardia autotrophica*, 99%, AP018920	**pos**	neg	neg	neg	neg	neg
